# Frequent epigenetic alterations in polycomb repressive complex 2 in osteosarcoma cell lines

**DOI:** 10.18632/oncotarget.25484

**Published:** 2018-06-05

**Authors:** Helin Feng, Heather Tillman, Gang Wu, Andrew M. Davidoff, Jun Yang

**Affiliations:** ^1^ Department of Surgery, St Jude Children’s Research Hospital, Memphis, TN 38105, USA; ^2^ Department of Pathology, St Jude Children’s Research Hospital, Memphis, TN 38105, USA; ^3^ Department of Computational Biology, St Jude Children’s Research Hospital, Memphis, TN 38105, USA; ^4^ Department of Orthopedics, The Fourth Hospital of Hebei Medical University, Shijiazhuang 050011, Hebei, People’s Republic of China

**Keywords:** polycomb repressive complex 2, EZH2, osteosarcoma, H3K27me3

## Abstract

Osteosarcoma (OS) cell lines are widely used in understanding the biological functions of cancer, identification and validation of therapeutic targets, as well as *in vitro* or *in vivo* preclinical drug screening. Here we report there is a frequent loss-of-function of polycomb repressive complex 2 (PRC2) in OS cell lines but it is rare in tumor samples based on genomic sequencing data, western blotting and immunohistochemistry analysis of H3K27me3. U2OS and 143B cell lines have a complete loss of function of PRC2 and several others have partial loss. In OS tumor tissues, only 1 out of 14 has low expression of H3K27me3. Kaplan-Meier analysis indicates that high EZH2, the component of PRC2, is associated with poor metastasis-free survival. Our observations are to raise the alarm that particular caution should be taken when using OS cell line models to study the disease, functional genomics, therapeutic target validation, drug screening, and epigenetic studies. Nevertheless, these cell lines will become useful biological tools to dissect the functions of PRC2.

## INTRODUCTION

Osteosarcoma (OS) is the most prevalent primary bone tumor and has a high propensity to metastasize. Although the 5-year survival rate has increased from about 20% to 65–70% after the introduction of chemotherapy in the 1970s, no significant improvements have been made since then [1, 2]. Therefore, there is a pressing need to develop new agents for OS therapy. Although it is well known that genetic features of cell lines may not represent those of primary tumor cells *in vivo*, established OS cell lines are widely used in understanding the biological functions of cancer cells, identification and validation of therapeutic targets, as well as *in vitro* or *in vivo* preclinical drug screening. For example, U2OS is one of the most frequently used cell lines in cancer biology research and Google Scholar had at least 29,000 records for this cell line. Twenty years ago, cancer biologists realized that gene expression and phenotype, including drug response, vary greatly due to culture conditions and passage numbers. These differences are one explanation for variations in biological processes in the *in vitro* and *in vivo* settings, which causes difficulties for data reproducibility. Here we report a frequent loss-of-function of polycomb repressive complex 2 (PRC2) in OS cell lines. This observation appears to be a rare event in primary tumor samples, and therefore may be a unique feature of some OS cell lines.

PRC2 consists of 4 core proteins (EZH2, SUZ12, EED, and RbAp46/48) and epigenetically silences gene expression by adding repressive histone methyl marks on lysine 27 of histone H3 (H3K27) [3]. Although each component of PRC2 is important for its function, the histone methyltransferase EZH2 is responsible for the catalytic activity. PRC2 is involved in various biological processes such as proliferation, differentiation, cell identity maintenance, and stem cell plasticity [3]. Overexpression of EZH2 promotes neoplastic transformation of normal prostatic cells and hyperplasia in the breast epithelium [4, 5], enhances tumor angiogenesis [6], invasion, and metastasis [7–9]. The importance of PRC2 in cancer has been further demonstrated by next generation sequencing of clinical cancer samples. Recurrent somatic mutations of EZH2 have been identified in subtypes of lymphoma, and EZH2 gain-of-function mutations alter substrate specificity for promoting hypertrimethylation of H3K27 [10]. However, the identification of loss-of-function mutations in PRC2 components in leukemia and in malignant peripheral nerve sheath tumors indicates that it also has tumor suppressive functions [11]. In addition, either gain or loss of function of PRC2 may lead to therapeutic resistance under different cellular context [12–16].

## RESULTS

**Figure 1:**
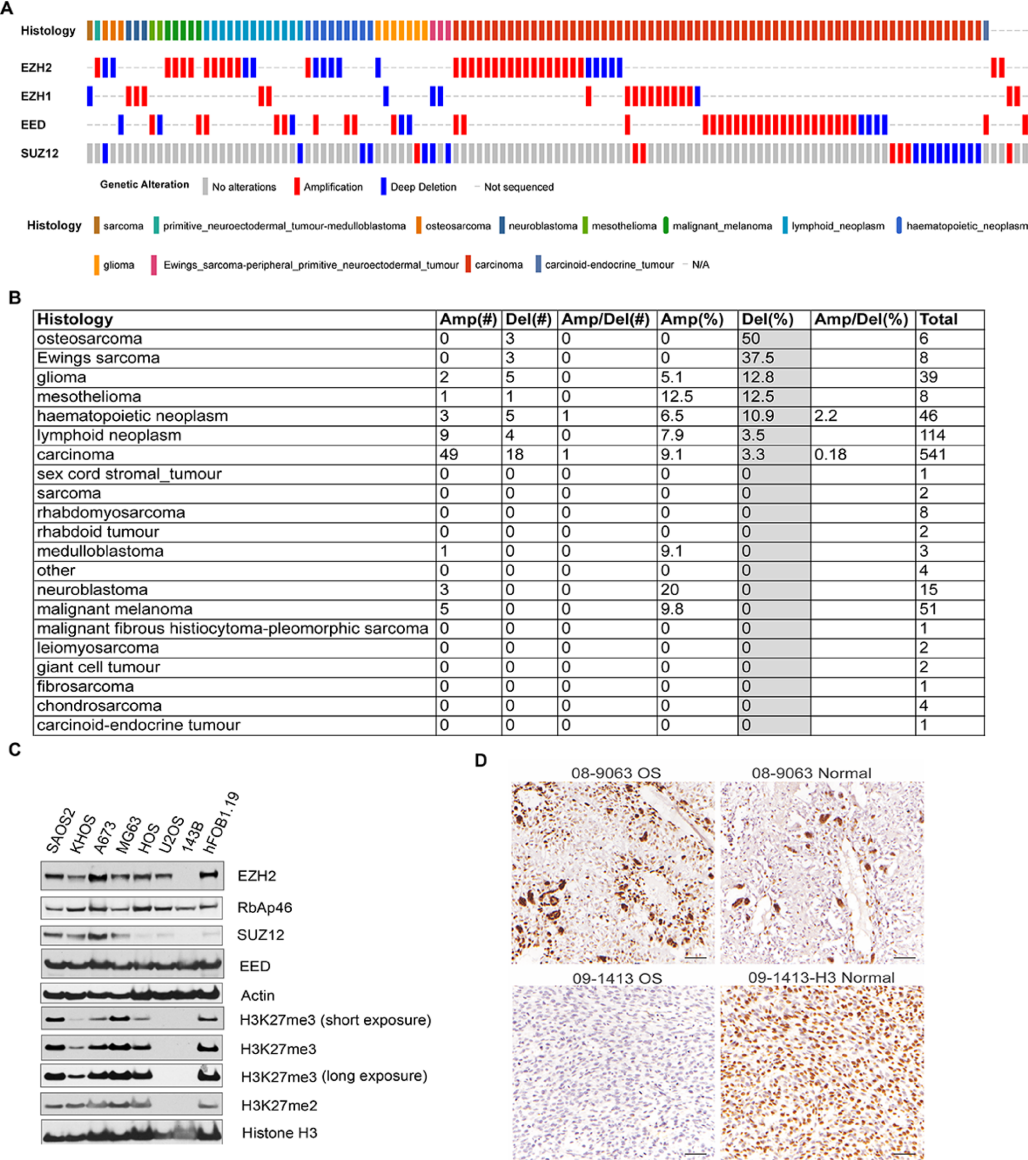
Genetic alterations of PRC2 components in osteosarcoma cell lines (**A**) The genetic amplification and deletion of PRC2 components in different histology cancers in CCLE data. The putative passenger mutations were not shown. (**B**) The percentage of genetic amplification/deletion of PRC2 components in different histology cancers in CCLE data. (**C**) Western blotting assessment of PRC2 complex and its epigenetic marks H3K27me2/me3 in osteosarcoma cell lines (SAOS2, KHOS-240S, MG63, HOS, U2OS, 143B), Ewing sarcoma cell line A673, and osteoblast cell line hFOB1.19 that was transfected with SV40 large T antigen. (**D**) Immunohistochemistry staining of H3K27me3 in 2 OS and adjacent normal tissues.

We have found a frequent loss-of-function of polycomb repressive complex 2 (PRC2) in OS cell lines after checking the genomic alterations of the cancer cell lines in Cancer Cell Line Encyclopedia(http://www.cbioportal.org) [17, 18]. Three out 6 of OS cell lines had genetic deletion in PRC2 components (Figure 1A), including HOS, U2OS and HS_888_T while MG63, SJSA-1 and T1-73 did not. Compared with other cell lines, OS cell lines ranked highest with 50% bearing genetic deletions of PRC2, while Ewing sarcoma cell lines were just below OS with 37.5% bearing genetic deletions of PRC2 (Figure 1B). Other lineages either had low frequencies of genetic amplifications or deletions of PRC2. We further examined the PRC2 function by Western blotting assessment of H3K27me3 in several OS cell lines and found that PRC2 had lost its function partially or completely in most of them (Figure 1C). The 143B cell line (>22,500 reference records on Google Scholar) has lost key components of the PRC2 complex including EZH2 and SUZ12, resulting in a total loss of H3K27me3/me2 marks (Figure 1C). While the U2OS cell line appears to maintain expression of EZH2 from one allele, it has completely lost the ability to catalyze H3K27, suggesting an unidentified mechanism that modulates PRC2 activity is dysfunctional. Interestingly, the PRC2 complex in KHOS-240S and HOS cell lines are also dysfunctional by being unable to catalyze H3K27me3, although H3K27me2 is not remarkably affected (Figure 1C). Although it is not new cancer cells frequently have genetic alterations, our data clearly showed that only OS cell lines but not other lineages had genetic and protein deficiency in PRC2 complex, which is unique and important.

Recent genomic sequencing data from our institute and other studies show that osteosarcoma rarely has genetic alterations in the PRC2 complex (Supplementary Table 1) [19–21]. In our dataset, we found only EZH1, a paralogue of EZH2, had an in-frame deletion in 1 out of 23 cases although its impact on H3K27me3 was not assessed [21]. In Perry et al study, SUZ12 was found to have copy number loss and somatic mutation in only 1 out of 59 cases [20]. In Behjati et al study, EED was found to have mutation in 1 out 112 tumors [22]. However, in Kovac [19] and Chiappetta [23] studies, no genetic alterations were identified in 92 tumors and 8 tumors, respectively. To assess H3K27me3 in tumor tissues, we performed immunohistochemistry for H3K27me3 in 14 cases of OS, 3 of which had adjacent normal tissue (Figure 1D and Supplementary Table 2). We found that nearly all osteosarcoma tissues bear detectable H3K27me3 (>50% nuclear positivity, Supplementary Table 2). However, one case showed low H3K27me3 levels compared with the adjacent normal tissue (15% vs 78.7% nuclear positivity, Figure 1D). These data seemed to be correlated with the RNA-seq data from OS tumor samples in that all tumors had detectable expression of PRC2 components [21] (Supplementary Figure 1). Although the clinical significance of H3K27me3 and PRC2 expression in OS needs to be further investigated by using a large cohort of cases, high *EZH2* gene expression appeared to be correlated with a poor outcome in osteosarcoma (Supplementary Figure 2).

## DISCUSSION

Despite the importance of PRC2 in cancer, functional studies of PRC2 in osteosarcoma are scarce. Nevertheless, our finding does not mean that the loss-of-function of PRC2 complex does not happen *in vivo* in OS. Although it is likely that the genetic/epigenetic loss-of-function of PRC2 in OS cells is due to selection under culture stress or intrinsic genetic instability, there is a third possibility that these cells were derived from rare clones that expanded when the cell lines were established using bulk tumor cells, especially for samples taken from relapsed, metastasized, or refractory disease. In the future, whether PRC2 is involved in metastasis of osteosarcoma warrants further studies. Our observations suggest more validation studies should be done when using OS cell line models to study the disease, functional genomics, therapeutic target validation, drug screening, and epigenetic studies.

Cell lines without detectable H3K27me3 have rarely been reported. Our discovery that OS cell lines have PRC2 function defects indicate these cell lines might be useful tools for further dissection of regulatory mechanisms of PRC2 mediated and independent functions. EZH2 has been reported to act as a transcriptional coactivator for androgen receptor [24], which is independent of its role in PRC2-mediated transcriptional repression. This functional switch is dependent on phosphorylation of EZH2 and requires an intact methyltransferase domain. Interestingly, recent studies have shown that EZH2 is subject to phosphorylation during G2/M phase of cell cycle by CDK1 and CDK2 [25–27], suggesting EZH2 function is incorporated into cell cycle regulation. EZH2 is able to methylate STAT3 to enhance its activity in glioblastoma stem cells [28, 29]. Yet EZH2 also has been shown to have methyltransferase activity-independent function in stabilizing BubR1 [30], a spindle assembly checkpoint protein, during mouse oocyte meiotic maturation. These studies further highlight that PRC2 and EZH2 regulation is highly context specific. Therefore, these unique cell lines may provide better insight into the novel biological functions of EZH2 or PRC2.

## MATERIALS AND METHODS

Please see Supplementary Materials.

## SUPPLEMENTARY MATERIALS FIGURES AND TABLES



## Abbreviations

PRC2: polycomb repressive complex 2
OS: osteosarcoma
H3K27me3: trimethylation of lysine 27 on histone H3
