# Supplementary material for: Frequent epigenetic alterations in polycomb repressive complex 2 in osteosarcoma cell lines
Source: Oncotarget. 2018 Jun 5;9(43):27087–91. doi: 10.18632/oncotarget.25484 (PMC6007463; doi:10.18632/oncotarget.25484)
Supplement: Supplementary file 1 [file oncotarget-09-27087-s001.pdf]

Frequent epigenetic alterations in polycomb repressive complex 2 in osteosarcoma cell lines

SUPPLEMENTARY MATERIALS

METHODS

Cell culture

Osteosarcoma cell lines SAOS2, KHOS-240S, MG63, HOS, U2OS, 143B, Ewing sarcoma cell line A673, and osteoblast cell line hFOB1.19 were purchased from ATCC. Cells were cultured in DMEM medium, supplemented with 10% FCS (HyClone, Thermo), 1% L-glutamine (MediaTech), 1% penicillin–streptomycin (Lonza). All cell lines were used under passage 5, characterized by Short Tandem Repeat (STR) analysis by using Promega PowerPlex 16 HS System. PCR based method was used for detection of Mycoplasma with LookOut Mycoplasma PCR Detection Kit (Sigma) and JumpStart *Taq* DNA Polymerase (Sigma).

Western blot

Cell lysates were either prepared using RIPA buffer (Cell Signaling) supplemented with protease inhibitors (Roche complete mini), followed by 5 min heating at 75° C after addition of 2 × Sample Loading buffer (100 mM Tris-HCl pH 6.8, 4% SDS, 20% Glycerol, 5% β-mercaptoethanol, 0.2% Bromophonol blue). Samples were separated on 4–12% Tris-glycine SDS-PAGE gels (Invitrogen), and transferred to PVDF membrane (Millipore). Membranes were blocked for 1hr in TBS buffer with 5% milk and 0.1% Tween 20, followed by overnight incubation with primary antibodies at 4° C. Membranes were washed for 3 × 5 min at room temperature in TBS-T buffer. Mouse and rabbit HRP-conjugated secondary antibodies (ThermoFisher, 1:5,000) were incubated for 1 hr at room temperature followed by washing 3 × 5 min at room temperature in TBS-T. For detection, membranes were exposed to Pierce ECL Western Blotting Substrate and detected on Hyblot CL film (Denville Scientific Inc). All the western blot antibodies are listed in the Key Resource section. Antibodies for EZH2, SUZ12, RbAp46, H3K27me3, H3K27me2, Histone H3 were purchased from Cell Signaling. EED antibody was purchased from Millipore. Actin antibody was purchased from Sigma.

Immunohistochemistry of H3K27me3

The osteosarcoma specimens collected at The Fourth Hospital of Hebei Medical University were fixed in 4% paraformaldehyde and embedded in paraffin. Sections (6 µm) were incubated with anti-H3K27me3 (1:200 dilution, Cell Signaling), following with secondary sheep-anti-rabbit antibody (1:1000 dilution, Dako, UK). Staining was visualized with an EnVisionTM Peroxidase/DAB Rabbit/Mouse detection kit (Dako, UK).

Microarray data mining

The expression of EZH2 from an osteosarcoma cohort (GSE42352) was used in Kaplan-Meier survival curve analysis using R2 program (http://r2.amc.nl). The median value of EZH2 expression was used for the cut-off level of EZH2 expression in survival analysis.

Genetic data mining

The genetic alteration data of PRC2 components were mined by using cBioportal program (http://www.cbioportal.org/).

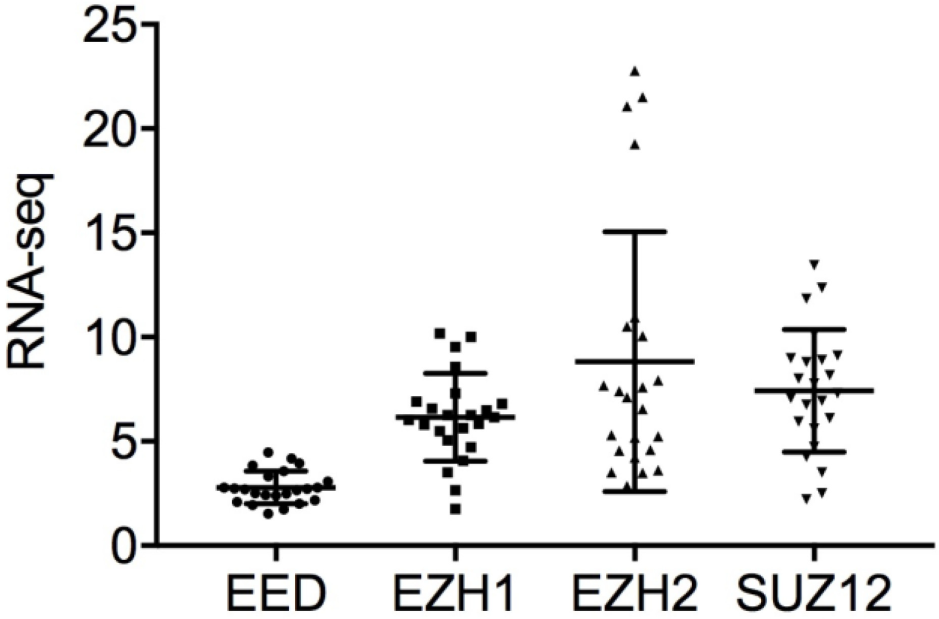

Supplementary Figure 1: PRC2 expression in an osteosarcoma cohort by Chen.

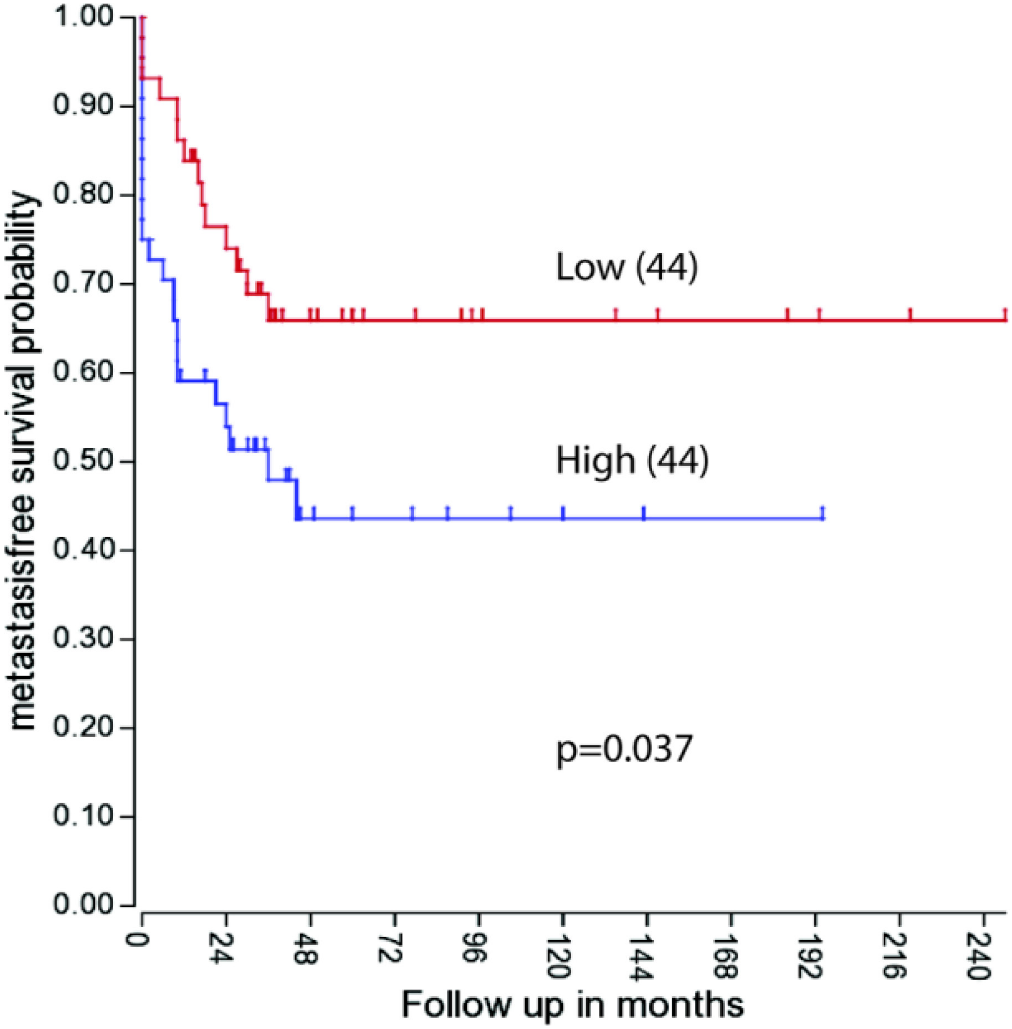

Supplementary Figure 2: Kaplan–Meier curve for EZH2 expression in an osteosarcoma cohort. The median expression value of EZH2 is used as cut-off from the dataset (Kuijier, GSE42352).

Supplementary Table 1: Genetic alterations of PRC2 components in 5 different cohort studies

	mutation type	EZH1	EZH2	SUZ12	EED	RbAP46/48
**Kovac data**	CNV	0	0	0	0	0
(92 tumors)	SNV	0	0	0	0	0
**Perry data**	CNV	0	0	1	0	0
(59 tumors)	SNV	0	0	1	0	0
**Chen data**	CNV	0	0	0	0	0
(34 tumors)	SNV	1	0	0	0	0
**Behjati data**	CNV	0	0	0	0	0
(112 tumors)	SNV	0	0	0	1	0
**Chiappetta data**	CNV	0	0	0	0	0
(8 tumors)	SNV	0	0	0	0	0

Abbreviations: CNV = copy number variation.
SNV = somatic nucleotide variation.

Supplementary Table 2: Score for immunohistochemistry staining of H3K27me3 in primary OS tissue sections

Slide label	Area analyzed (mm2)	Total nuclei	H3K27me3 (+) nuclei	% positive cells	Positive cells/ mm2 tissue	Tissue
05-15983	22.8	68533	45867	66.9	2007.8	OS
06-11281A 16-11-10	29.0	71744	54975	76.6	1898.0	OS
06-13934A H3 16-11-10	8.8	54127	46314	85.6	5269.6	OS
06-15778 16-11-5	30.4	107666	87309	81.1	2868.2	OS
06-23146A 16-11-6	19.7	61565	43845	71.2	2228.6	OS
08-1066 A 16-11-10	1.2	5514	4190	76.0	3451.4	OS
08-9063 16-11-6 #1	40.4	186611	150924	80.9	3735.2	OS
08-9063 16-11-6 #2	54.3	232727	102817	44.2	1892.6	Normal
08-15243A 16-11-10	11.9	64231	36602	57.0	3067.0	OS
08-16444 16-11-9	13.6	54631	44197	80.9	3255.8	OS
08-16669 16-11-10	4.7	26928	23296	86.5	5005.6	OS
09-1413 6 16-10-31	27.4	116630	18520	15.9	675.3	OS
09-1413 H3 16-10-31	49.6	283620	223418	78.8	4506.9	Normal
09-6077 10 16-11-5	2.1	5452	3882	71.2	1850.4	OS
09-6077 16-11-5	25.5	97442	81721	83.9	3199.0	Normal
09-6883B 16-11-10	29.3	89772	86914	96.8	2969.9	OS
09-14231A H3 16-10-31	34.6	142984	78519	54.9	2269.1	OS
